# Low frequency water level correction in storm surge models using data assimilation

**DOI:** 10.1016/j.ocemod.2019.101483

**Published:** 2019-12

**Authors:** Taylor G. Asher, Richard A. Luettich Jr., Jason G. Fleming, Brian O. Blanton

**Affiliations:** a Department of Marine Sciences, University of North Carolina, Chapel Hill, NC, United States of America; b Institute of Marine Sciences, University of North Carolina at Chapel Hill, Morehead City, NC, United States of America; c Seahorse Coastal Consulting, LLC, Morehead City, NC, United States of America; d Renaissance Computing Institute, University of North Carolina, Chapel Hill, NC, United States of America

## Abstract

Research performed to-date on data assimilation (DA) in storm surge modeling has found it to have limited value for predicting rapid surge responses (e.g., those accompanying tropical cyclones). In this paper, we submit that a well-resolved, barotropic hydrodynamic model is typically able to capture the surge event itself, leaving slower processes that determine the large scale, background water level as primary sources of water level error. These “unresolved drivers” reflect physical processes not included in the model’s governing equations or forcing terms, such as far field atmospheric forcing, baroclinic processes, major ocean currents, steric variations, or precipitation. We have developed a novel, efficient, optimal interpolation-based DA scheme, using observations from coastal water level gages, that dynamically corrects for the presence of unresolved drivers. The methodology is applied for Hurricane Matthew (2016) and results demonstrate it is highly effective at removing water level residuals, roughly halving overall surge errors for that storm. The method is computationally efficient, well-suited for either hindcast or forecast applications and extensible to more advanced techniques and datasets.

## Highlights

• A water level data assimilation method for coastal hydrodynamic models is developed.
• The method is tested with 3 sources of meteorological forcing for Hurricane Matthew.
• The method roughly halves modeled storm surge errors.
• It corrects for background water levels and errors in far-field meteorology.
• It increases computational burden by roughly 2–8% in operational forecasts.

## 1 Background

The hazards of coastal flooding have been observed repeatedly throughout human history. 49% of deaths from Atlantic hurricanes in the U.S. (1963–2012) have come from storm surge (Rappaport, 2013). Hurricane damages in the U.S. (1900–2005, from all sources) average roughly $10 billion dollars per year (Pielke et al., 2008). In extreme cases, direct economic damages have exceeded $100 billion (Hurricane Harvey (2017), Hurricane Katrina (2005) and the Great Miami Hurricane (1926)), and death tolls have extended into the hundreds of thousands (Bhola cyclone (1970), Cyclone Nargis (2008), and others). Enhancing the predictive power of storm surge models is therefore of great importance.

Improvements in forecasting and hindcasting of tropical cyclones (TCs) have led to much more accurate meteorological data associated with these storms (Cangialosi, 2018, Landsea et al., 2012). These improvements, together with more detailed representations of the physics and greater model resolution, have improved storm surge model skill (Resio and Westerink, 2008). This has led to a state in which storm surge forecast and hindcast errors are often dominated by what we refer to as *unresolved drivers*, i.e., physical processes that are not explicitly included in storm surge models. Common examples include baroclinic processes, major oceanic currents, precipitation, steric fluctuations, and far-field atmospheric forcing. These phenomena typically have longer timescales than the storm surge itself and therefore often show up as gradually varying residuals in water levels.

This paper presents a method for improving coastal water level predictions in hydrodynamic models through assimilation of water level data to correct for errors introduced by unresolved drivers. The method was developed to improve multi-day coastal water level forecasts, and is shown herein to be highly effective for retrospective studies, as well. The paper’s structure is as follows: The remainder of Section 1 provides background on errors in forecasts, the nature of unresolved drivers, and a brief overview of data assimilation (DA) techniques and their use in surge models. These ideas are combined to argue in favor of a specific combination of DA and model techniques in Section 2, the details of which are then presented in Sections 3, 4. A case study demonstrating the effectiveness of this approach is shown using Hurricane Matthew (2016) in Sections 5, 6. We then discuss justification, limitations, and alternatives for the proposed DA framework in Section 7. Discussion of the method’s use in an operational forecast setting follows in Section 8, before concluding. Data and simulations presented in this study have been published (Asher, 2019) and made publicly available online through DesignSafe-CI at https://doi.org/10.17603/2Z8H-7K90.

### 1.1 Advances in surge modeling

Uncertainties in meteorological forecasts are often the leading-order source of uncertainty in storm surge forecasts of tropical cyclones: The 2017 24-, 48-, and 72-h track errors for tropical cyclones in the Atlantic basin are 32, 56.4, and 87.7 nautical miles (59.3, 104, 162 km), respectively (Cangialosi, 2018). These distances can constitute the difference between peak and relatively minor surge. However meteorological forecast errors continue to decrease, creating a critical window 1–3 days prior to landfall (and, roughly, peak storm surge) when surge model errors due to factors other than meteorological forecast uncertainty are becoming significant in the overall predictive power of storm surge forecasts.

**Fig. 1:**
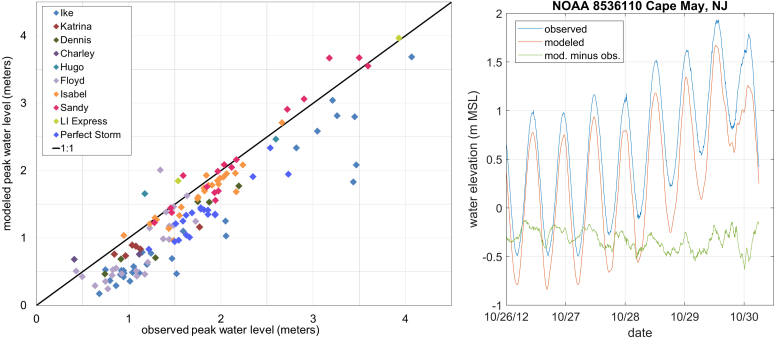
Characteristic storm surge model error. Left: Modeled vs. observed peak surges when mean water level offsets are not accounted for, all points are gage data. Right: Modeled and observed water levels during Hurricane Sandy (2012) at the NOAA Cape May, NJ gage 8536110.

State of the art storm surge models utilize the shallow water equations, often in depth-integrated, barotropic form. They include forcing from tides, wind and atmospheric pressure, bottom drag, and in some cases wind waves. The history, resolution requirements, and improvements in storm surge models have been characterized in several studies, including Bode and Hardy (1997), Blain et al. (1998), Dukhovskoy and Morey (2011), and Kerr et al., 2013b, Kerr et al., 2013a. Model skill has improved notably over the past two decades due to improvements in computational power, topographic data quality, meteorological model skill, and model physics. For instance, DA in meteorological model hindcasts produces wind and pressure fields that more closely match actual events and significantly improves storm surge model skill (Cardone and Cox, 2009). Surge models are now reaching a state where low-frequency variations in water levels can be a primary contributor to error, if not accounted for, as shown in Fig. 1. These data come from validation hindcasts with the ADCIRC hydrodynamic model (described in Section 5.1) during development of the National Oceanic and Atmospheric Administration (NOAA) HSOFS mesh (Riverside Technologies, Inc., and AECOM, 2015). Simulations were run with model zero at mean sea level to match anticipated operational conditions, and the resulting low bias is apparent. The difference in modeled and observed mean water level and inspection of time series data (right Fig. 1), make clear that, although higher frequency fluctuations are evident, slowly-varying changes in the water elevation drove much of the low bias for nearly all storms. Typically, surge hindcasts either de-trend measured data to remove such “background” water levels, or apply a spatiotemporally constant water level offset to the model that approximates the mean water level preceding the storm in the vicinity of maximum surge (e.g. Westerink et al., 2008). However, this fails to account for spatial variations, which may be appreciable depending on the unresolved driver(s) responsible and the size of the area affected by surge. In operational forecast applications, changing the water level correction between simulations could apply a shock to the model, and if spatially varying, would create associated large-scale flows, making it more suitable as a post-processing step. But post-processing means physical effects of the altered water level (and therefore depth) are not included in the model simulation, and also creates challenges in changing the horizontal extent of the calculated floodplain after the simulation completes, which can be quite complicated during major surge events.

Data from Riverside Technologies, Inc., and AECOM (2015).

### 1.2 Unresolved drivers

Hydrodynamic models designed to accurately simulate storm surge must resolve domains covering thousands of kilometers and features such as elevated roadways and dunes only several meters wide (e.g. Bilskie et al., 2015). This results in the need for substantial computational resources, and a desire to optimize model complexity. Thus, storm surge models are typically depth-integrated, constant density, and decoupled from large scale oceanic motions, rainfall, and hydrologic input (except at relatively large scale). In the case of TCs, since gridded meteorological data may not adequately resolve the storm’s core, storm surge models often use parametric representations of the storm’s wind and pressure fields (such as Holland (1980)), thereby neglecting both antecedent and far-field meteorological conditions. These introduce a host of unresolved drivers with varying temporal and spatial scales. For instance: (1) seasonal water level fluctuations have a spatial scale of hundreds to thousands of kilometers and a temporal scale of months to a year (e.g. Enfield and Allen, 1980); (2) fluctuations in major ocean currents extend hundreds to thousands of kilometers and for days to years (Meinen et al., 2010, Valle-Levinson et al., 2017); (3) runoff from major rainfall events may affect tens to hundreds of kilometers and lasts several days to weeks. Notably, these processes are slow compared to storm surge, particularly due to tropical cyclones.

#### 1.2.1 Magnitudes

Unresolved drivers generally produce smaller coastal water level fluctuations than tides and storm surge. Seasonal water level fluctuations (U.S. monthly averages available from https://tidesandcurrents.noaa.gov/sltrends/sltrends.html) often exceed 10 cm along the open coast (e.g. Barbosa et al., 2008). The rise in coastal water levels following a rainfall event usually comes after storm surge, with magnitudes of ∼10 cm or less typical during the surge event, though as evidenced in North Carolina during Hurricane Florence (2018), notable exceptions to this may occur in semi-enclosed coastal water bodies. Interannual anomalies in coastal water levels on the order of ∼5 cm have also been detected and related to local and broader atmospheric forcing patterns (Andres et al., 2013, Calafat et al., 2018, Hong et al., 2000). Changes in Gulf Stream transport have been implicated in coastal sea level fluctuations of up to several 10 s of cm (Ezer and Atkinson, 2017, Goddard et al., 2015, Sweet et al., 2009). Atmospheric systems can reduce Gulf Stream transport by ∼30% (Mooers et al., 2005); during the time period preceding and including Hurricane Matthew, the Florida current transport dropped by approximately 30% (Fig. 4 left). Ezer and Atkinson (2017) associated a 30–40 cm rise in coastal water levels following the passage of Hurricane Matthew with the large drop in Gulf Stream transport.

### 1.3 Efforts in ocean data assimilation

Popular DA methods include Kalman filter variants (Verlaan and Heemink, 1997, Evensen, 2003, Altaf et al., 2014), optimal interpolation (OI) (Oke et al., 2002, Counillon and Bertino, 2009a, Madsen et al., 2015), and variational methods (Bennett, 2002, Kurapov et al., 2011, Troupin et al., 2012). OI typically uses the variability in the observations and an error covariance matrix, assumed a priori, to create a spatial field that blends the model and observations, and can emulate physical behavior by selecting appropriate covariance function(s) (e.g. Cooper and Haines, 1996, Lorenc, 1981). OI is actually a sub-optimal version of the more general Kalman filter, and more advanced Kalman filter-based approaches do away with (parts of) these a priori assumptions, using a variety of techniques to better propagate the error covariance. These and advanced variational methods come at greater computational cost than OI due to the complexity of the analysis and/or the need to run multiple model realizations.

DA in coastal water level problems is not new (e.g. the storm surge work of Heemink, 1986), and has been successfully employed in the North Sea (e.g. Zijl et al., 2015). Storm surges in this region are often large (geographically) and remotely forced (Madsen et al., 2015). Combined with the area’s large tidal fluctuations and dense sensor network, DA is able to correct errors introduced by the use of a coarse numerical model (e.g. Høyer and Andersen, 2003). Efforts in other regions have shown some promise: Etala et al. (2015) demonstrate short-term (6 h) forecasting improvements using satellite altimetry and coastal water level data along the Argentine coast, and multiple authors have successfully calibrated models via assimilation (e.g. Graham et al., 2016, Mayo et al., 2014). However, tropical cyclones’ strong local forcing and smaller spatiotemporal scales create many challenges for practical data assimilation. Butler et al. (2012) and Altaf et al., 2013, Altaf et al., 2014 improved hurricane surge prediction with a very coarse surge model using several different ensemble Kalman filters. However, major gains were only seen for a very short (2-h) forecast window and required careful tuning of the assimilation scheme. Improvements more than 2 h out were much more modest, and required a set of several hundred synthetic water level observations across the continental shelf in the vicinity of the storm track (Fig. 4, Fig. 5 in Butler et al., 2012), a dataset which does not yet exist. Butler et al. also noted no improvement in forecasts longer than 36 h for H. Katrina, whereas H. Ike showed some improvement at 48 h. We interpret this as being due to the exceptionally long surge signal seen preceding H. Ike, due to the storm’s massive size and unusual forerunner surge (Kennedy et al., 2011). Similarly, Peng and Xie (2006) noted no improvement in storm surge forecasts beyond 8 h using 4DVar to revise initial conditions (with a coarse 3D ocean model). By also revising the wind drag coefficient with 4DVar (and the same model), Li et al. (2013) realized moderate improvements when wind speed was deliberately set too high/low, though the choice of correcting wind speed error with drag coefficient is fortuitous. These cases point to a fundamental limitation in utilizing DA for improving storm surge forecasting that will be explored in the next section as we assess what sort of DA might best benefit this problem.

## 2 Requirements for storm surge data assimilation

Both the physics model and the DA method face choices that balance complexity and computational cost: Forecast coastal water level modeling systems must consider the relative importance of model physics, grid resolution, the number of forecast ensemble members, timeliness, etc., given available computational resources. Advanced DA methods, such as 4DVar or ensemble Kalman filters, require substantial computational resources, sometimes over an order of magnitude greater than simulations without assimilation. Conversely, the a priori assumptions made in OI typically lead to lower-quality results at a lower computational burden.

A second consideration is the timescale of the surge event itself. Peak surge events from TCs often last on the order of 12 h (e.g. Hurricanes Matthew in Fig. 8, Fig. 9, and Floyd in Fig. 10), forecast cycles occur every 6 h, and lead times for emergency preparation and evacuation are up to several days. *The time scale of significant storm surge is shorter than the lead time needed for action*; storm surge is often a locally driven process, which can mean water level data with a strong surge signal cannot be assimilated early enough to provide useful information. This point is particularly important, as it possibly cannot be overcome through more data, computational resources, or DA methods. Similarly, DA cannot be expected to represent phenomena whose spatial scale is smaller than the spacing between observations (typically 10–200 km along the U.S. Gulf and Atlantic coasts).

Given the phenomenological and computational constraints, the question becomes: how can one most effectively use DA to improve storm surge forecasts? We argue that a low-cost DA method paired with existing surge model physics is an effective balance. Our approach is to use DA to address the water’s response to more slowly varying, large scale unresolved drivers, while using the hydrodynamic model to accurately capture the generation and propagation of surge and tides.

## 3 Assimilation formulation

In this section, we describe our approach for ingesting water level corrections into the model, while the next section steps through how the correction is generated and implemented in practice. We are working with the Reynolds averaged, depth integrated, constant density shallow water equations (Vallis, 2006, Luettich and Westerink, 2004),

(1) $$\begin{array}{c} \text{continuity}:\\ \frac{D\left(h+\zeta \right)}{Dt}+\left(h+\zeta \right)\nabla \cdot U=0\\ x\;\text{momentum}:\\ \frac{DU}{Dt}-fV=-g\frac{\partial \zeta }{\partial x}-\frac{1}{\rho_{0}}\frac{\partial p}{\partial x}+\frac{\tau_{sx}-\tau_{bx}+\tau_{wx}}{\left(h+\zeta \right)\rho_{0}}+\frac{\partial \alpha \eta }{dx}+M_{x}\\ y\;\text{momentum}:\\ \frac{DV}{Dt}+fU=-g\frac{\partial \zeta }{\partial y}-\frac{1}{\rho_{0}}\frac{\partial p}{\partial y}+\frac{\tau_{sy}-\tau_{by}+\tau_{wy}}{\left(h+\zeta \right)\rho_{0}}+\frac{\partial \alpha \eta }{dy}+M_{y} \end{array}$$

for *h* the static water depth, ζ the water surface elevation, *t* time, *D/Dt* the material derivative, **U** the depth-averaged velocity vector (*U*, *V*), *f* the Coriolis parameter, *g* acceleration due to gravity, *p* pressure at the sea surface, $\rho_{0}$ constant water density, subscripts *x* and *y* indicating terms in their respective directions, $\tau_{s}$ surface wind stress, $\tau_{b}$ bottom drag stress, $\tau_{w}$ wave radiation stress, η Newtonian equilibrium tidal potential, α effective earth elasticity factor, and *M* vertically-integrated lateral stress gradient. The continuity equation imposes a mass balance on the solution while the momentum equations impose the force balance.

Our goal is to apply a gradually varying (in space and time) correction to the water elevation field, Δ ζ. A direct manipulation of the water level will not accomplish this by itself because a rapid barotropic adjustment would occur in the velocity field and the added water would simply flow away. Rather, we found this could be done effectively by introducing a fictitious force into the momentum equations to drive the solution to the desired surface elevation. Specifically, changes in atmospheric pressure create corresponding changes in water surface elevation (e.g., the bulge in sea level under an atmospheric low-pressure center). This can be approximated via the inverse barometer relationship: Δ*p* = − $\rho_{0}$ *g* Δ ζ; we therefore modify the sea surface pressure term in the momentum equations to reflect both the actual atmospheric pressure and a pseudo atmospheric pressure:

(2) $$\begin{array}{cc} p & =p_{a}+\Delta p\\ & =p_{a}-\rho_{0}g\Delta \zeta \end{array}$$

where $p_{a}$ is the actual atmospheric pressure and Δ*p* is a so-called pseudo atmospheric pressure (PAP). Considering only the x-momentum equation yields:

(3) $$\frac{DU}{Dt}-fV=-g\frac{\partial \zeta }{\partial x}-\frac{1}{\rho_{0}}\frac{\partial \left(p_{a}+\Delta p\right)}{\partial x}+\frac{\tau_{sx}-\tau_{bx}+\tau_{wx}}{\left(h+\zeta \right)\rho_{0}}+\frac{\partial \alpha \eta }{dx}+M_{x}$$

Substituting in Eq. (2) and regrouping terms, this can also be written as:

(4) $$\frac{DU}{Dt}-fV=-g\frac{\partial \left(\zeta -\Delta \zeta \right)}{\partial x}-\frac{1}{\rho_{0}}\frac{\partial p_{a}}{\partial x}+\frac{\tau_{sx}-\tau_{bx}+\tau_{wx}}{\left(h+\zeta \right)\rho_{0}}+\frac{\partial \alpha \eta }{dx}+M_{x}$$

In either form, a fictitious force is being applied to drive are-arrangement of the total water elevation field to include the assimilated elevation differences. The resulting adjustment of the total water elevation field then propagates through all other terms in the shallow water equations.

While adding the fictitious forcing term will drive water to achieve the desired surface elevation correction over time, we can assist this process by simultaneously introducing the desired water level correction directly into the water elevation field. As noted above, the elevation field cannot be modified without the compensating forcing term in the momentum equations because the added water would quickly flow away. When directly applying a correction Δ ζ to the water elevation, the correction in the momentum equations (Eq. (4)) can be thought of as providing a force to maintain the elevation correction.

Using only the fictitious force term, we are altering the velocity field to accomplish the re-arrangement of water to achieve the desired total water elevation. Adding water in directly minimizes the need for this dynamic re-arrangement and therefore lessens the accompanying velocity field alteration. However, under the assumption that the corrections are gradual in space and time, the two methods should exhibit similar behaviors, which is what we observed in practice in open coastal waters. Differences were primarily located in areas with significant constrictions. The similarities and differences between the methods are discussed in more detail in Section 7.4. For the sake of clarity in the remainder of the manuscript we term the PAP method as applying only the fictitious forcing in the momentum equations while the CON method applies the forcing and directly adds/removes water.

## 4 Storm surge data assimilation framework

**Fig. 2:**
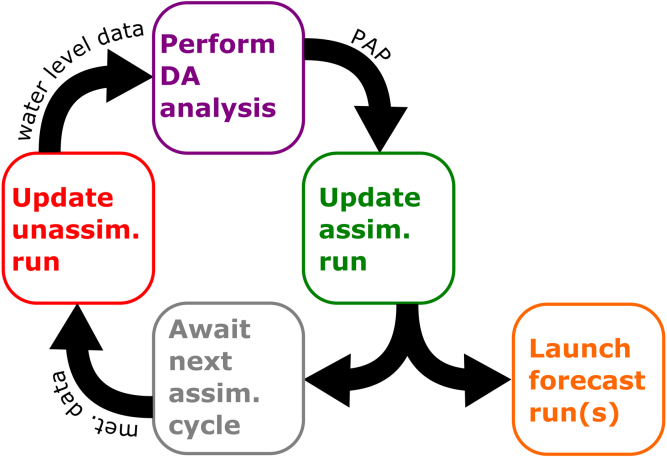
Schematic representation of the DA system; Supplemental Figure 1 provides a detailed schematic.

The DA system employed here can be broken down into four steps, (1) performing an unassimilated simulation, (2) time-averaging or lowpass filtering the difference between this simulation and observed water levels at observation sites, (3) generating a spatial difference field from these differences, (4) repeating the simulation with the added correction applied in the model. Fig. 2 shows a simplified schematic of the process with steps 2–3 lumped together; a more detailed schematic is shown in Supplemental Figure 1. The procedure is repeated at selected intervals in time, e.g., every forecast cycle in a forecast system, and the PAP forcing is interpolated in time between cycles. The unassimilated and assimilated model states are saved at the end of each simulation so that they may be resumed at the start of the next cycle.

The DA framework used here can be classified (e.g. Daley, 1991) as a continuous sequential scheme using optimal interpolation, in that the DA analyses are carried out every assimilation cycle (“sequential”) and the correction is applied continuously to the simulation (“continuous”) by way of the PAP forcing (and the mass field, if desired). Several parts of our approach are similar to those developed in the DA literature, including the time-distributed averaging procedure of Oke et al. (2002) and the incremental analysis update technique of Bloom et al. (1996). In the remainder of this section, we describe methodological details of the 4 steps; a step-by-step summary is provided alongside Supplemental Figure 1. We provide a more thorough discussion of our choices and alternatives in Section 7. Before proceeding, however, we make two clarifications on nomenclature. First, in DA literature, “forecast” often refers to an unassimilated system state, such as an unassimilated simulation; the term “background” is also used for this quantity, though its history is somewhat complicated (see Ide et al., 1997), so here we use “first guess” for this quantity to avoid confusion with a true forecast. Second, we use the term “difference field” here to refer to the spatial field of differences between observed and unassimilated modeled water levels.

### 4.1 Step 1: Unassimilated simulation

The “first guess” field of water elevations is determined by running an unassimilated simulation, whose final state is saved to permit restarting at the beginning of the next assimilation cycle. Separating the unassimilated and assimilated simulations is unusual in DA (we term this a “two-track” assimilation scheme), however it ensures that our model solution is driven to match the missing low frequency component without introducing overshoot and undershoot oscillations.

### 4.2 Step 2: Time-averaging

The “first guess” and the observed water level data are averaged over a chosen time period *T* (nominally 1–10 days) in order to filter out high frequency fluctuations, such as astronomical tides, that constitute most of coastal water level variations. Selecting the time scale as an integer multiple of the dominant tidal cycle (12.42 h for M2 semidiurnal tides) suppresses tidal phasing errors that might cause undesired oscillations in the difference field. Further discussion on the choice of time scale is presented in Section 7.3.

### 4.3 Step 3: Difference field

The mean difference between observed and modeled data is then fed into OI with an assumed error covariance to produce a gridded difference field (e.g., Fig. 6). Calculating and storing the full-resolution covariance matrix, a 10^6^ × 10^6^ matrix for the 1.8 million-node mesh used in this study, would be a daunting task, and it is common in the DA literature to partition the domain into subdomains to manage this problem. However, we argue the time-averaging of water levels, the gradual nature of the unresolved drivers we wish to capture (compared to typical grid resolutions), and the large distance between observations make the use of a coarser grid for data assimilation computations appropriate, after which results are interpolated to the model mesh.

To explain generation of the difference field, we begin with a brief introduction to sequential data assimilation. There is substantial variability in the notation utilized in DA literature, however we mostly follow that of Ide et al. (1997). The base equation in sequential data assimilation is,

(5) $$\begin{array}{c} x^{a}=x^{f}+K\left(y^{o}-Hx^{f}\right)=x^{f}+\mathrm{Kd}\\ d=y^{o}-Hx^{f} \end{array}$$

where bold lowercase/uppercase variables denote vectors/matrices. We now define the variables and their dimensions in context of traditional DA terminology (in quotes); their meaning for the problem at hand is supplied in the following paragraph.

*n* Number of grid points
*m* Number of observed values, denoted *p* in Ide et al. (1997)
**x**${}^{f}$=**x**${}^{f}$(*t*) (*n* × 1) Time-varying “forecast”, “background”, or “first guess” system state vector
**y**${}^{o}$=**y**${}^{o}$(*t*) (*m* × 1) Time-varying “observation” vector
**x**${}^{a}$ (*n* × 1) The “analysis” vector
**K** (*n* × *m*) The (Kalman) “gain matrix” or “gridding operator”
**H** (*m* × *n*) The “observation operator” or “measurement operator”
**d** (*m* × 1) The “innovation vector” or “observational residual”

Put in the context of this problem, *n* is the number of grid points; *m* is the number of gages where observed water levels are available for assimilation; **x**${}^{f}$ are the water levels from an unassimilated model simulation at the grid points; **y**${}^{o}$ are the observed water levels at the gages; **x**${}^{a}$ are the water levels calculated by the assimilation process at the grid points at a single point in time; **H** linearly interpolates the unassimilated model water levels to the locations of the observations; **d** is a set of water level differences at the observed locations; and **K** performs the heavy lifting of creating the difference field **Kd**. In reality, **K** depends on **H** and the error covariance structure of the first guess and the observations. For a conceptual overview, readers are directed to a recent review article (Moore et al., 2019); for further details, interested readers are directed to Fletcher (2017), Bertino et al. (2003), Bennett (2002), and Evensen, 2003, Daley, 1991.

Calculation of the water level correction is termed the “analysis step” in DA. We do not directly apply Eq. (5) in our method. Rather, we time-average **d** as described in the last section and compute the difference field as,

(6) $$\Delta \zeta^{a}=K{\langle d\rangle }_{T}$$

for Δ $\zeta^{a}$ (*n* × 1) the difference field (corresponding to Δ ζ) and *T* the time scale of the averaging operator denoted by angle brackets. We then introduce this time-averaged difference field in the model to apply the correction gradually, and let the model’s physics respond accordingly, using either the PAP or CON methods, as explained in Section 3.

### 4.4 Step 4: Assimilated simulation and forecast

With a new difference field calculated, the assimilated simulation is then restarted from its last saved state and run forward from the previous cycle until the current time is reached, much like the unassimilated simulation in step 1. Each difference field is considered to represent the correction for the current time, and so the PAP forcing (and the elevation correction, if desired) applied during a simulation evolves in time by linearly interpolating between the previous and current difference fields. If this is used as part of a forecast system, the forecast simulations would then be initiated using that assimilated solution as the initial condition together with some assumption of how the difference field evolves into the future.

## 5 Case study setup

Modeling experiments were undertaken to evaluate the ideas presented here and to build an algorithm for carrying out such assimilations under operational conditions. Hurricane Matthew (2016) was selected due to the presence of a persistent bias between predicted and observed water levels prior to and throughout the event and the availability of high-quality data to support the investigations. The ADCIRC model, coupled with the SWAN wave model, was used. Data assimilation was done with water level data from a set of coastal water level gages. Each of these components is described in further detail in this section.

### 5.1 Numerical model

ADCIRC is an ocean circulation model based on the 2D/3D shallow water equations. It solves these equations in a continuous Galerkin, linear finite element, unstructured mesh framework (Luettich and Westerink, 2004, Westerink et al., 2008). Here, it is used in its 2D depth-integrated form. SWAN is a 3rd-generation phase-averaged spectral wave model that solves the conservation of wave action equation as it evolves in time and space (Booij et al., 1999, Zijlema, 2010). It operates on the same linear triangular mesh as ADCIRC. The two models are tightly coupled, with water depths and currents passed to SWAN and wave radiation stress gradients passed to ADCIRC at each shared time step (Dietrich et al., 2011b, Dietrich et al., 2011a). The coupled model is highly parallelized for execution on supercomputing systems (Tanaka et al., 2011, Dietrich et al., 2011a, Kerr et al., 2013a), making it useful for numerous surge modeling applications.

**Fig. 3:**
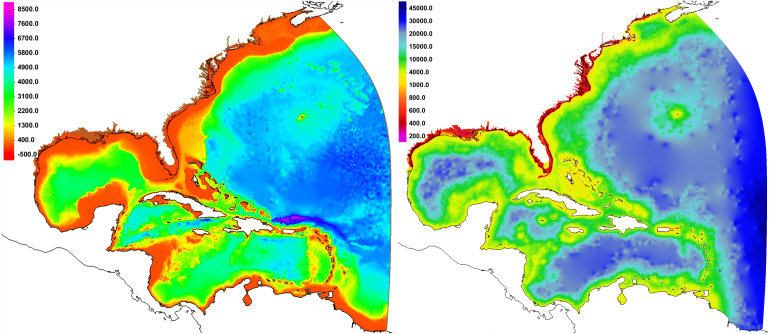
HSOFS mesh topobathy (left, meters below MSL) and node spacing (right, meters); black lines are coastlines, brown lines (left) are inland model boundaries and levees.

The 1.8 million node, 3.6 million element HSOFS mesh was used (Riverside Technologies, Inc., and AECOM, 2015). Shown in Fig. 3, the HSOFS mesh covers the western north Atlantic, and all overland areas of the neighboring U.S. coast up to an elevation of 10 meters above sea level, with detailed representations of inlets, rivers, barrier islands, roadways, and other key features. Elevation data is based on the latest available bathymetry and lidar-derived topography. Mesh resolution in coastal areas is typically 400 to 500 meters (and as low as 200 meters in some areas), making it an intermediate-resolution grid developed specifically for national-scale real-time storm surge forecasting applications. This mesh is now used by NOAA for operational coastal water level and storm surge guidance in their ESTOFS (Extratropical Surge and Tide Operational Forecast System) and HSOFS (Hurricane Surge On-demand Forecast System) programs.

Meteorological forcing is shared by ADCIRC and SWAN and is described in the next section. Tidal forcing in the ADCIRC model is specified by periodic water elevations along the offshore boundary and body forcing throughout the domain based on the Oregon State University TOPEX/Poseidon Global Inverse Solution version 7.2 (TPXO7.2) (Egbert and Erofeeva, 2002). Manning’s n-based bottom friction in ADCIRC is based on 2006 C-CAP land cover data, so that increased dissipation during flooding in marshes and overland areas is accounted for. Directional reductions in wind speed due to the roughness of overland features is accounted for using the land cover data upwind of a given location to determine the roughness height in a Charnock-type relation for Oceanweather and Holland-type wind models (discussed in the next section), since these are considered to be at marine exposure. Overland drag for waves is considered less important since their primary use here is in helping calculate accurate surge elevations via wave radiation stresses and depth-induced breaking is the primary mechanism of energy dissipation in such cases. Bottom friction in SWAN is formulated as a JONSWAP-type sea state-dependent drag (Hasselmann et al., 1973), and no wind reductions are performed. The ADCIRC model is explicit and finite difference in time, and so has a Courant-limited time step of 2 s for this application. The SWAN model is time implicit, allowing it to have a larger 15-minute time step. More granular detail on model setup parameters is provided in the supplemental.

### 5.2 Hurricane Matthew

**Fig. 4:**
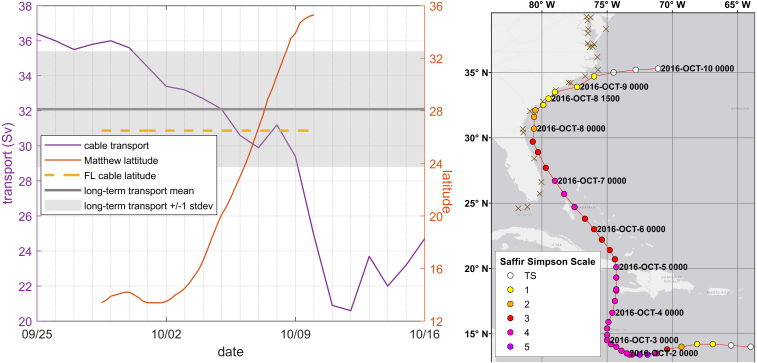
Hurricane Matthew and its effects on the Gulf Stream. Left: Florida current transport during Hurricane Matthew; orange lines denote the latitude of Matthew’s eye and the FL cable current measurement. Right: Gage sites and storm track; dots along Hurricane Matthew’s track denote 6-h interval positions and landfall, brown X’s denote gages in the assimilation set.

Hurricane Matthew was a Cape Verde type^1^ hurricane that developed into a tropical cyclone on September 28, 2016, near Barbados. The storm passed between South America and the Greater Antilles, briefly becoming a Category 5 hurricane on the Saffir Simpson hurricane wind scale, before turning north on October 2. It made landfalls on Haiti and Cuba on October 4 and 5, respectively. It then tracked just offshore of the southeastern U.S. coastline for several days, barely making landfall in South Carolina on October 8, before transitioning to an extra-tropical cyclone on October 9 and heading offshore (Stewart, 2017). Although the storm’s surges were not as severe as many other historic storms, Matthew’s track (Fig. 4) brought moderate surges to over 1000 km of U.S. coastline, and high water conditions for several days at many sites, providing a large amount of observational data over which to study the storm’s effects.

Meteorological forcing for the coupled model is provided in the form of winds and sea level pressures, and is taken from three sources in order to assess the effects of different meteorological fields. The most basic meteorological forcing comes from a parametric vortex model, the Generalized Asymmetric Holland Model or GAHM, based on Holland (1980), but without requiring the cyclostrophic balance assumption (Gao, 2018). Parametric models such as GAHM are useful in that they allow wind and pressure fields to be constructed in a dynamically consistent manner directly from meteorologist forecast parameters. In the case of GAHM, radii to maximum winds are determined in each storm quadrant by fitting the model to specified wind isotachs in each quadrant (Gao, 2018, Dietrich et al., 2018). There are no far field winds associated with the GAHM other than those of the translating vortex of the storm. An intermediate quality meteorological forcing was developed by combining the GAHM with North American Mesoscale Forecast System (NAM) fields, the latter supplying the far field winds and pressures. Blending between the two datasets is performed over a range of several radii to maximum winds away from the storm center in order to provide a smooth transition. The third and highest-quality source of wind and pressure forcing comes from reanalysis meteorological fields defined objectively using airborne, terrestrial, and oceanic measurements, including stepped frequency microwave radiometer (SFMR) data, by OceanWeather Inc. (OWI), (Cox et al., 1995, Cardone and Cox, 2009).

### 5.3 Observation data

Water level data was collected from a total of 44 permanent stations along the open coast, estuaries, bays, and rivers, and 129 temporary storm tide sensors deployed for Hurricane Matthew. All of these data are from either NOAA or the U.S. Geological Survey (USGS). 24 NOAA gages were used in the assimilation set that forms the **y**${}^{o}$ observations needed for DA (shown in Fig. 4). 19 gages and 129 storm tide sensors (visible in Fig. 7) were used as validation of the scheme. The final gage, located at Springmaid Pier, SC, was lost when the pier was destroyed by Matthew shortly before the storm surge peaked. This gage was also included in **y**${}^{o}$ (m=25) while functioning, but then dropped (m=24) when found to be out of operation, without any apparent loss of fidelity in the solution, suggesting the methodology is reasonably robust to events like gage failures, provided sufficient gages are included in determining the difference field. All gages in the assimilation set were located at or close to the open coast, whereas the validation data are generally in more sheltered waters, rivers, and overland. Further information on all data used is supplied in Supplemental Table 2.

### 5.4 Data assimilation

DA was carried out using the method described in Section 4. Assimilated simulations were run using both the PAP method and the CON method as described in Section 3. A 6-h assimilation cycle was used that corresponded to the hurricane advisories provided by the National Hurricane Center, (Fig. 2). Every assimilation cycle, the first guess field **x**${}^{f}$ was updated with a 6-h model run, using the ending state of the previous unassimilated solution as its initial condition. Observed and unassimilated modeled water levels were averaged over the previous 24.84 h (two M2 tidal cycles) and were used to compute differences at the gage locations. These differences were input into OI assuming a Gaussian error covariance matrix with a constant correlation length^2^ of 1 degree. In addition, the difference field was constrained to approach zero offshore by placing a sequence of artificial “gages” just off the continental shelf break, with their **d** set to zero. Offshore of these areas, the difference field was discarded. An example of a resulting difference field for each of the meteorological forcings is shown for 00:00 h UTC on October 5, 2016 in Fig. 6. A 6-h model run with assimilation was then performed, using the ending state of the previous assimilated solution as its initial condition, and linearly interpolating the difference field in time from the previous to the newly constructed difference field over the 6-h assimilated model run. If this were to be used as part of a forecast modeling system, the assimilated model run would then be used as the initial condition for the forecast.

## 6 Case study results

Simulations were carried out from 00:00 UTC October 2 to 00:00 UTC October 11, 2016 with the three sources of meteorological forcing both with and without assimilation, giving a total of 6 simulations for hurricane Matthew. Runs without assimilation are referred to as “baseline” cases, and the two-letter shorthand nomenclature shown in Table 1 will now be used to refer to individual simulations. Minimal differences in skill metrics were observed between the results from the PAP and CON methods and so only the PAP method results are presented in this section; CON results are in supplemental material. Data and simulations presented here are available online through their publication (Asher, 2019).

### 6.1 Model skill

**Fig. 5:**
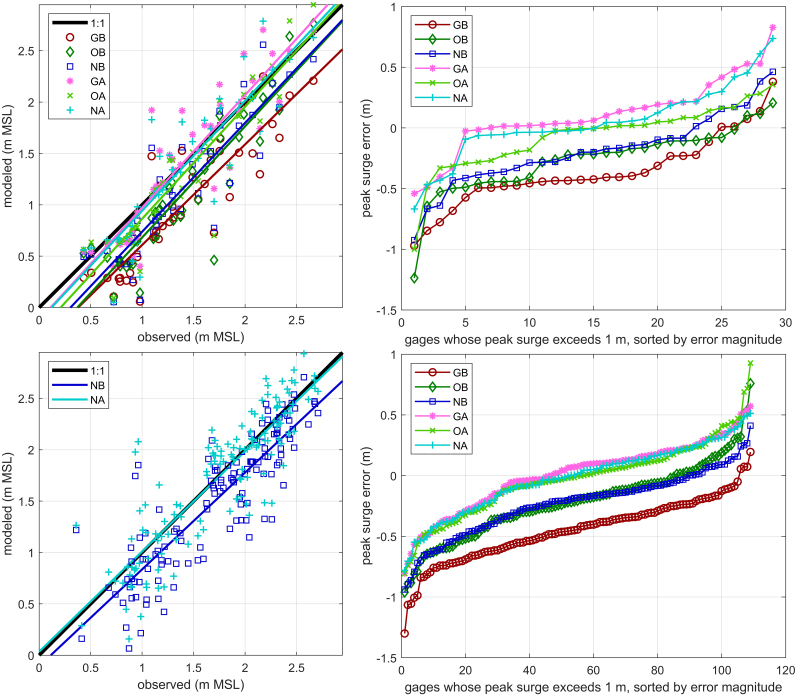
Comparison of peak modeled and observed surges. Top: assimilation sites. Bottom: Validation sites. Left: colored lines are linear fits. Right: only gages whose observed peak surge exceeded 1 m are displayed to filter out non-event data. A matching figure for the CON method is in Supplemental Figure 2.

Errors in peak surge estimates are provided in Fig. 5, Fig. 7; time series at selected gages are shown in Fig. 8, and time series plots at all gages are in Supplemental Figure 3. Skill metrics for both peak and time series data are provided in Table 1, with bias being positive when the model water level is higher than the observed. Time series statistics were calculated over the 120 h from October 5 00:00 to October 10 00:00 UTC.

All three baseline simulations show negative (model underestimation) biases, ranging from −22 to −79cm at the 24 assimilation sites. The GB simulation has the greatest bias, being approximately twice that of the OB and NB simulations, which have similar biases. With DA, the bias is largely removed, ranging from 1 cm to −6cm, as measured by peak surge or time series across all assimilation and validation stations for the three meteorological forcings. Mean absolute error (MAE) and root mean square error (RMSE) are reduced by a quarter to two thirds of their baseline simulation values as well. Error statistics for the CON method (Supplemental Table 1) were within 1 cm of the PAP method, with no systematic trend.

The largest remaining errors in the DA simulations occurred within Pamlico Sound and its accompanying rivers, in North Carolina. These errors are due to a combination of a frontal weather system north of Matthew that is not well represented in any of the meteorological forcing datasetsas well as errors in model bathymetry within the Sound. Model simulations (not shown) using a different grid with higher resolution and better bathymetry gave an increase in peak surge of 30 cm at the NOAA Hatteras gage (Fig. 8), bringing it into much closer agreement with observations.

**Table 1:** Simulation identification and skill metrics for peak and time series water level data at assimilation sites and for peak data at validation sites, in meters. A matching table for the CON method is in Supplemental Table 1.

**Fig. 6:**
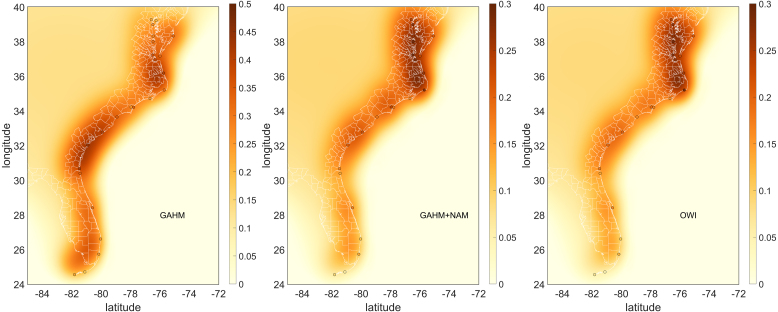
Difference fields (colors are in meters) along the U.S. Atlantic coast on Oct. 5, 2016 00:00 UTC for the three meteorological forcings, circles denote assimilation sites and their differences. Note the different elevation scales for the panel corresponding to the GAHM forced solution and the other two solutions.

**Fig. 7:**
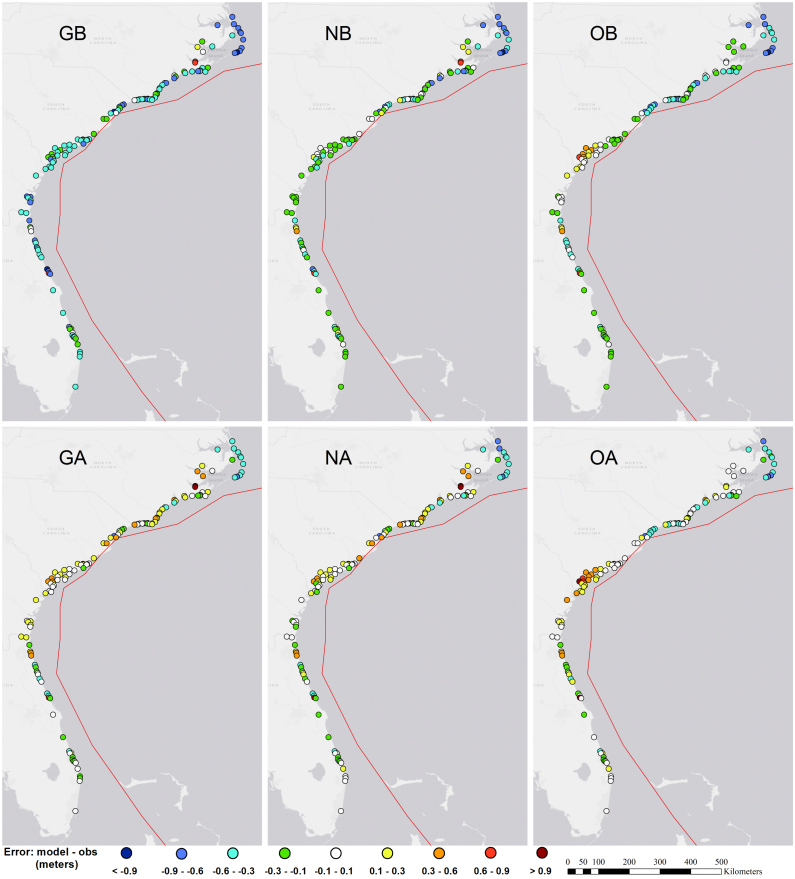
Error in peak surge at storm tide sensors, modeled minus observed, meters; left-to-right: GAHM winds, OWI winds, GAHM+NAM winds; top: baseline simulations; bottom: simulations with assimilation. Red line is Hurricane Matthew’s track.

**Fig. 8:**
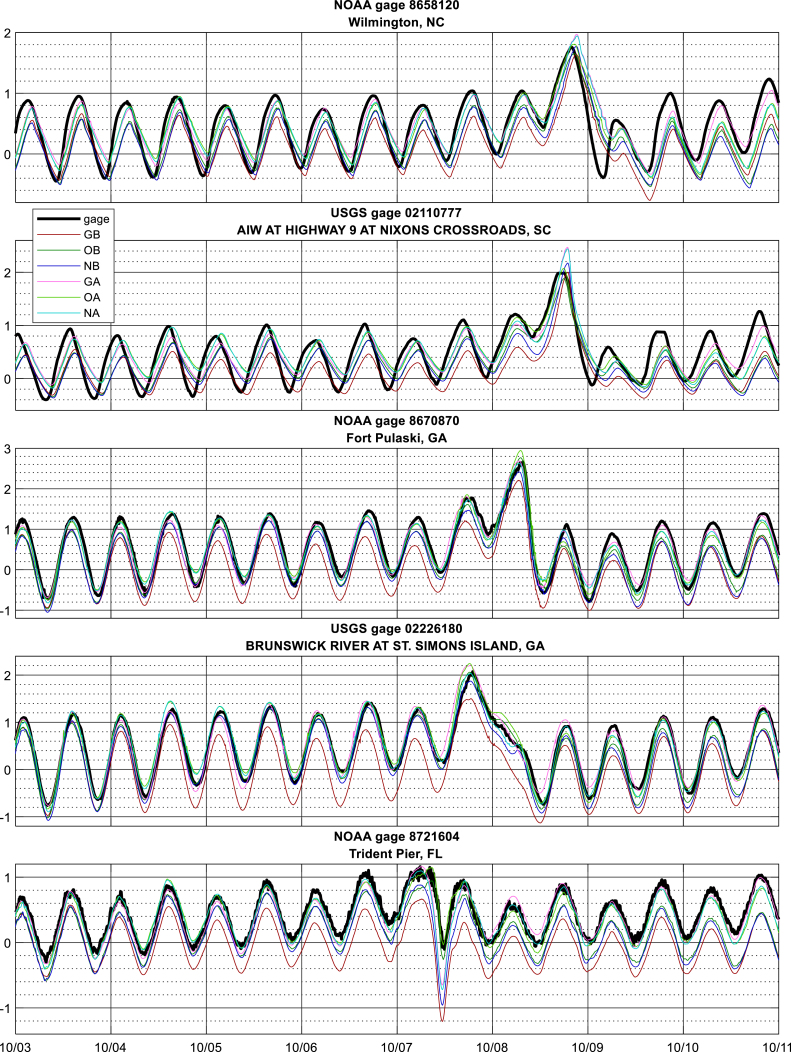
Time series of modeled and observed water levels (m MSL) at select gages; black (gray) titles denote gages in (not in) the assimilation set.

**Fig. 9:**
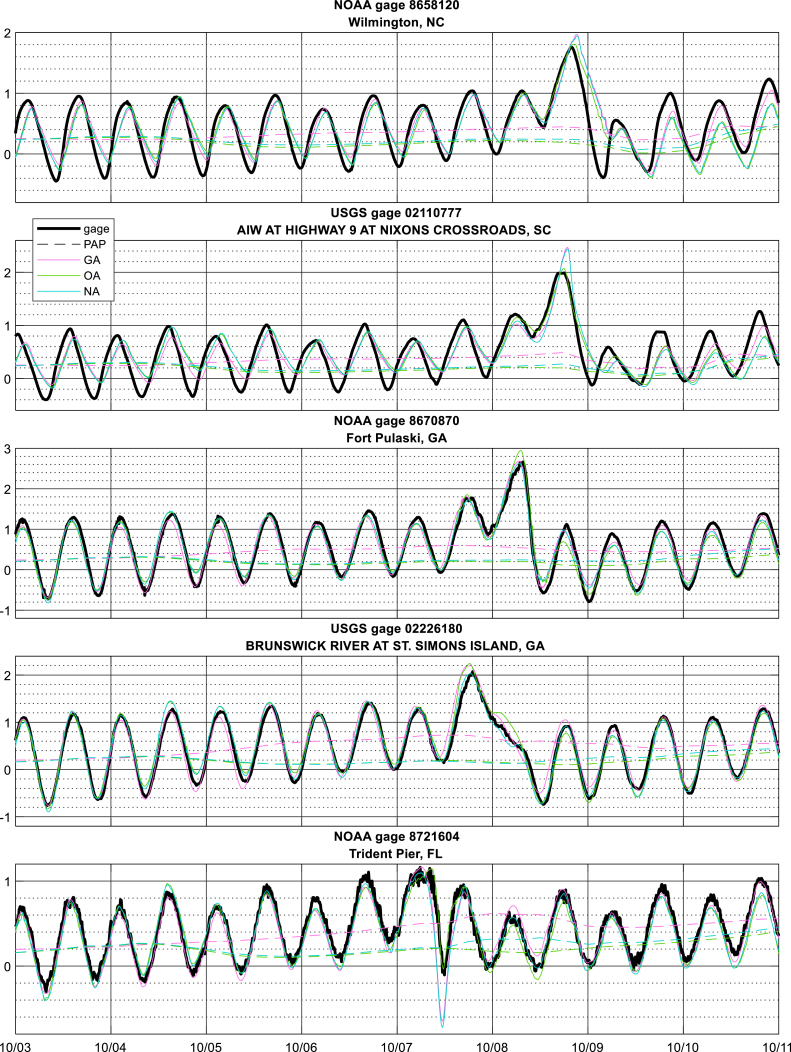
Time series of modeled and observed water levels (m MSL) at select gages, PAPs (units of meters water) denoted by dashed lines in matching colors; black (gray) titles denote gages in (not in) the assimilation set.

### 6.2 Assimilation effects

The spatial structure of the difference fields indicates a slight increasing trend from south to north along the US southeast Atlantic coast, Fig. 6. At this time, 00:00 UTC 10/5/2016, the eye of Matthew was located at approximately 20 degrees North (Fig. 4). The temporal behavior of the difference field is shown at selected stations along the coast in Fig. 9, time series plots at all gages are in Supplemental Figure 3. The two M2 tidal cycle time average and the Gaussian error covariance with 1-degree correlation length together appear to impose reasonable spatial and temporal smoothness in the difference field, consistent with the objective of correcting for low frequency unresolved drivers. The difference field is generally twice as large for the GB as the OB and NB solutions, consistent with the increased bias noted above.

The substantial reduction of model bias (and accompanying other error statistics) in the DA simulations provides strong evidence that this approach is effectively correcting for the unresolved drivers in the water surface elevation. Comparison of the GB and NB simulations suggests that approximately half the bias in the GB simulation may be due to missing far field meteorology, and that DA is able to reasonably compensate for this deficiency in model forcing. Comparison of the OB and NB simulations suggests that the blended GAHM+NAM wind and pressure fields provide a reasonable forecast-grade substitute for the more detailed reanalysis OWI fields. Assuming that the OB and NB reasonably capture the meteorological component of the water level forcing, the remaining half of the bias in the GB run and the majority of the bias in the OB and NB runs must be due to non-meteorological unresolved drivers. Water levels are characteristically high in October, with historical monthly average water levels ranging from 19 cm at Daytona Beach, FL to 17 cm at Fort Pulaski, GA, to 12 cm at Beaufort, NC. The area from southern Florida to Cape Hatteras is adjacent to the Gulf Stream, which experienced a substantial decrease in transport during Matthew’s passage (Fig. 4). Decreased Gulf Stream transport has been associated with increases in coastal water level (Ezer and Atkinson, 2017), and this may provide an additional unresolved driver during the Matthew time period.

## 7 Data assimilation framework discussion

As presented above, we have found our DA approach to be an effective tool for reducing a significant source of error in storm surge simulations. Calculating a difference field (Eq. (6)) involves multiple choices including the form of the gain matrix **K**, the time-averaging used to calculate ${\langle d\rangle }_{T}$, and the *m* observation sites that form **y**${}^{o}$. Lastly, there is the matter of selecting how data are manifested, or “ingested” back into the model, as was done via the PAP forcing alone (PAP method) or the forcing paired with direct addition of water (CON method). Each of these items will now be addressed, both in terms of the proposed method and in terms of the techniques presented in the meteorological and oceanographic DA literature.

### 7.1 Gain matrix K

The most challenging step in determining **K** is defining the first guess error covariance matrix, typically denoted **B** or **P**${}^{f}$ (note the latter does not denote the PAP field). It is common to partition **B** into a diagonal variance matrix **D** (*n* ×*n*) that can optionally be updated in time and a stationary (i.e. time-independent) correlation matrix **C** (*n* × *n*), as $B=D^{1∕2}CD^{1∕2}$. We expect strong anisotropy in the correlation structure, with distinct characteristic scales in the cross- and along-shore directions, which should evolve through estuaries, bays, and further upstream. Therefore, presuming a single correlation length is clearly suboptimal, as indicated in the following example.

**Fig. 10:**
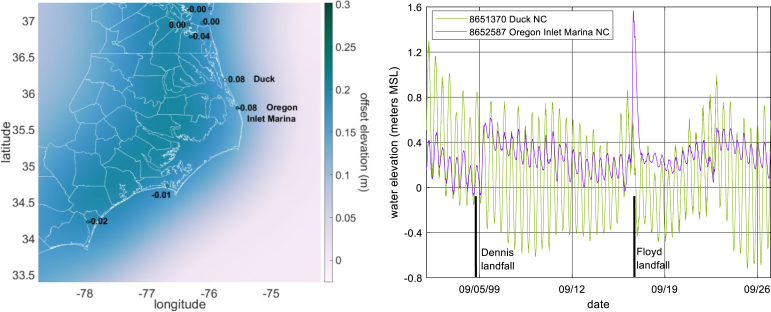
Water level anomaly around Hurricanes Floyd and Dennis. Left, the water level anomaly surface calculated for the 4 days prior to the landfall of Floyd, numeric labels indicate the difference (in meters) between the OI water level anomaly surface and the observed mean water level at the site; white lines are coastline and county boundaries. Right, water elevation time series at the two gages highlighted in the left panel.

In 1999, Hurricanes Dennis and Floyd dropped massive amounts of rain in North Carolina within a few weeks of each other, leading to record-breaking flooding across much of the state. This resulted in differences in water levels on either side of the North Carolina barrier island complex as high as 0.5 m. Fig. 10 shows the effect this had on the water level anomaly surface, determined via OI using a 4-day average water level prior to Floyd and a constant correlation length of 1 degree. The open-coast gage at Duck shows a small anomaly in the water level time series since it is not appreciably affected by the rainfall, however the gage at Oregon Inlet shows a much larger, longer-lasting anomaly due to the runoff filling up Pamlico Sound. The water level anomaly surface using our simple correlation structure is unable to match the 16 cm difference in the 4-day mean water level between the two gages. Application of a more appropriate correlation matrix would permit a sharp spatial gradient to be more faithfully^3^ represented in the difference field.

We expect that the structure of the correlation matrix **C** will depend on the unresolved driver(s) at work, and therefore it is unclear whether a single covariance structure can fully account for distinct phenomena. If a substantial portion of the to-be-assimilated model error **d** is presumed to come from physics absent from the model, and not errors due to model forcing, initial conditions, etc., then along with the sparsity of measurements, this means *a substantial portion of the error covariance structure is not identifiable*.^4^ Put another way, the correlation (on all time scales of interest) between the sheltered Oregon Inlet and open-coast Duck locations (discussed in the previous paragraph) can only be estimated with measured data or a model with the physics needed to resolve all time and space scales of interest. So, one must either rely on an incomplete estimate of **C** from the surge model’s correlation structure, or else infer it from another source, such as altimetry data, a more advanced model, or a parametric function. This difficulty in revealing the full covariance structure motivates a generalization of Eq. (6) to,

(7) $$\Delta \zeta^{a}=\overset{J}{\underset{j=1}{\sum }}K_{j}{\langle d\rangle }_{Tj}$$

where *j* is the index of *J* difference fields; note we will use $d_{j}$ as shorthand for 〈d〉 ${}_{Tj}$. This permits accounting for different unresolved drivers separately, each with its own correlation matrix $C_{j}$. With this, we can consider a series of spatiotemporal scales corresponding to different unresolved drivers, whose covariance structures can be inferred using one of the methods mentioned in the last paragraph. Eq. (7) is attractive because it conforms nicely to one interpretation of the theoretical basis for the forcing term, that it represents a closure scheme for a series of unresolved drivers, discrete physical processes that the hydrodynamic model’s governing equations and inputs do not explicitly account for.

While conceptually attractive, many physical processes have overlapping scales, making separation difficult to accomplish in practice. Thus the approach of defining different correlation matrices for different unresolved drivers may be most useful for separating out shorter fluctuations (e.g., days to weeks) from those that affect seasonal- or longer-term water levels, since correlation lengths for the latter processes are substantially longer (e.g. Calafat et al., 2018).

### 7.2 Sites *m*

Greater data density and quality generally results in improved estimates. However, since the physical dynamics are not accounted for in our implementation of OI, utilizing data which are not representative of the coastal environment under consideration can be problematic if the data are not handled with care: Preliminary tests utilizing water level gages in rivers were found to produce lower quality difference fields because our OI implementation does not resolve the pathway taken by the water, and so the area of influence of these gages is exaggerated. This, like the example in Section 7.1, may be alleviated through use of proper correlation structure.

Being unbound by physical constraints, the difference field can diverge when extrapolating, which is why we imposed the artificial “gages” (with their **d** set to zero) off the continental shelf break to weakly constrain the difference toward zero in the offshore direction, as discussed in Section 5.4. Physical constraints could be applied to deal with such issues within the DA scheme, however the irrelevance of such small offshore water level fluctuations on coastal water levels suggests any technique may suffice. A similar issue arises in inland areas, where the **d** at the coast may not be relevant. If inland gages are not available or used, then in cases where the inland domain extent is large compared to the correlation length, ways to constrain the difference field inland may need to be devised.

### 7.3 Time scales T

Time-averaging in calculating $d_{j}$ acts as a boxcar filter on the water level difference. It is not clear the choice of averaging method is particularly important, and other analyses have shown that simple filtering methods can be advantageous (Bloom et al., 1996, Oke et al., 2002). However, the choice of averaging time scale *T* is key. The exact choice of time scale represents a balance between which fluctuations to remove and which to keep, and so can depend on the processes of interest. Use of a (rather short) 24.84-h averaging period in this study permitted DA to correct for the surge error resulting from poor far-field meteorology in the GAHM model, however no systematic study was undertaken to evaluate whether this was the best choice of *T*. Review of time series in Fig. 9 suggests an averaging period 1.5x or 2x this length may have also produced similar results, though this question may warrant further review under other conditions.

Our approach may create a lag in the modeled response since (1) the difference field is based on data prior to the current point in time, and (2) there may exist some lag time in the model responding to the forcing in the case of the PAP method. Lag due to the former should be small unless **d** shifts rapidly compared to the assimilation interval. Lag due to the latter is discussed in Section 7.4. Note that lag due to the former issue could also be resolved by centering the assimilation window in time.^5^

### 7.4 Ingestion method

Techniques for incorporating the water level correction back into a model simulation, which we term “ingestion”, are built into methods like certain ensemble Kalman filters and 4DVar, one of their advantages; there is no such prescribed method for OI. We proposed two methods in Section 3. The Matthew case study did little to elucidate differences, as the methods’ error statistics were nearly identical. Therefore, we now present a simple test problem, and use it to distinguish the methods and how they each modify the solution.

The GA simulation presented in Section 6 was repeated, with all forcing disabled except for the assimilation to isolate its effects. The GA simulation’s difference field was chosen because it is a realistic test case with relatively large elevations, peaking at almost 0.7 m in some regions, and rising/falling up to 20 cm/day. 9-day PAP and CON simulations were conducted, with the initial elevation difference ramped in over 31 days (prior to the beginning of the 9-day simulation) to ensure both simulations start at the same equilibrium state. Note that simulation days 31–40 here correspond to October 2–11, 2016 in the Matthew case study.

**Fig. 11:**
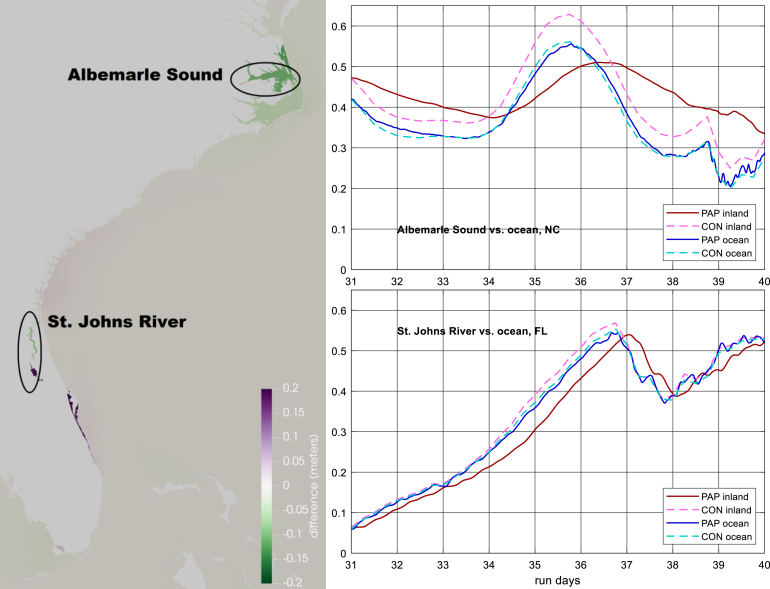
Differences in simulated water levels between the CON and PAP methods. Left, the difference in water level (CON minus PAP, meters) at 37.25 days (corresponding to 6:00 AM UTC October 8th). Top right, water level time series (meters MSL) in Albemarle Sound and at the nearby open ocean. Bottom right, water level time series (meters MSL) in the St. Johns River and at the nearby open ocean.

Results are shown in Fig. 11. Across the entire study area, maximum differences in the two simulations are less than 2 cm except for three regions, ordered from north to south: The Pamlico-Albemarle Sound complex in North Carolina, the St. Johns River in Florida, and the Indian River Lagoon system in Florida. In these areas, differences reached 5–20 cm. These areas are characterized by having tightly restricted flows to the ocean. In all cases, the CON method’s water levels track the difference fields more closely than the PAP’s. This is expected given that the PAP method requires the elevation field to respond to the imposed forcing whereas the CON method imposes the elevation change directly. However, which result is preferable is situational.

In open coastal areas and areas with moderate connections to the open coast, the propagation is fast and the difference between the methods minimal (Fig. 11). However, the PAP method fills and empties tightly restricted semi-enclosed waterbodies more slowly. For example, the Pamlico-Albemarle Sound complex, a very large bay with very narrow openings, filled at 8–15 cm/day during the PAP assimilated simulations. Noting that the correction field is derived primarily from coastal gages, during Matthew this may reflect a realistic damping of the coastally-derived elevation correction. This suggests the relative utility of the two methods is largely relegated to waterbodies with a weak connection to the open ocean and is tied to the expected accuracy of the difference field, i.e., does the difference field correctly represent the water elevation difference inside these waterbodies (in which case the CON method may be more accurate) or is it simply a landward extension of the coastal water level difference (in which case the PAP method may be more accurate).

One case of special note is a fully enclosed waterbody, such as a coastal pond or gated waterway. The CON method would raise/lower water levels in the waterbody whereas the PAP method would only impose a gradient across the waterbody until the water level in an adjacent coastal region rose to a level that caused it to become connected with the enclosed waterbody. Here, again, which result is preferable depends on one’s expectation of the true difference field.

## 8 Operational considerations

Operational forecasts carry substantially different priorities from those of other modeling studies. In the former, a critical requirement is getting an answer in a timely fashion. To the degree the quality of the answer can be improved, it should be, but subject to the constraints that the improvement can be computed quickly enough to allow the result to be timely and that the process does not cause the forecast to fail (e.g., introduce model instability).

The need to perform two simulations, one with and one without assimilation means that this method roughly doubles the computational requirements for a hindcast study; calculation of the difference field itself takes minimal time. However, in an operational forecast setting, this doubling only applies to the simulation period going from past to present (Supplemental Figure 1), typically a 6-h window. The result of the assimilation is the creation of an improved initial condition for the impending forecast(s). Assuming a 6-h assimilation-forecast cycle, the computational burden increases by 7.6% for a single 3-day forecast or 4.6% for a single 5-day forecast. This small computational burden decreases even further if a multiple forecast ensemble is computed using the same initial condition (e.g., to 1.6% for a 3-day, 5-member ensemble forecast). Since computing the difference field does not require the forecast meteorology, it is possible to run the unassimilated simulation and compute the difference field concurrent with the generation of the meteorological forecast. If this is done, the additional computational time required to implement DA in the storm surge model would not delay the surge forecast (which must wait for the forecast meteorology to become available).

The reliability of the difference field is a function of the quality of the input data as well as the choices made in computing it. It is therefore important that any observation data stream be quality-controlled. The failure of the Springmaid Pier gage during Matthew is an example of the loss of a real-time data stream. The OI surface in this case study did not change markedly after the gage failed and was removed from the data used to determine the difference field, as seen in the time series in Fig. 9 at the nearby Oyster Landing gage. The Charleston SC and Wrightsville Beach NC gages are the two nearest to Springmaid Pier, each roughly 1 degree away. This indicates the 1-degree correlation length is reasonably chosen, given the density of available observations and the smoothness of the assumed Gaussian covariance. Operational applications will need to choose sites and covariance structure in a way that ensures loss of a gage (or gages) does not lead to physically unrealistic difference fields.

When carrying out a true forecast, some assumption must be made about how the difference field transforms into the future. In our initial use of this approach in forecast applications we have held the difference field constant through the forecast cycle. More sophisticated approaches could be proposed incorporating expected growth/decay times implied by the characteristic time scale of the dominant unresolved driver(s).

## 9 Conclusions

Storm surge modeling systems have grown in complexity and accuracy, and have reached a state where unresolved drivers are major contributors to model errors. Several such drivers are well known to cause appreciable coastal water level variations. Changes in large-scale currents like the Gulf Stream are of particular curiosity, since they have been shown to be affected by passing storms, and there is recent evidence suggesting this could be an important component of multi-day water level fluctuations not reproduced by surge models. Hurricane Matthew appears to be one example of this.

Tropical cyclone-driven surge itself is often a rapid response that is not well suited for DA in forecasts and seems better handled by the physics of the model itself. In particular, there appears to be a limit to how much DA can improve tropical cyclone surge forecasts because guidance is often needed earlier than meaningful water level data can be assimilated. Conversely, many unresolved drivers typically vary over spatial and temporal scales longer than that of storm surge, and are therefore good candidates for DA to allow allocation of computational resources elsewhere. To this end, a system for assimilating water level data into a surge prediction system has been constructed, based around the principle of low-cost, continuous sequential data assimilation. It was implemented as a pseudo atmospheric pressure forcing term with and without a direct correction to the water elevation term in the ADCIRC surge model, and was evaluated for the case of 2016’s Hurricane Matthew using multiple meteorological data sources. Comparing results from the GAHM parametric vortex model with the blended GAHM+NAM model suggests that approximately half of the low bias in the GAHM simulation was due to missing far field winds. Counter to prior experiences, high-grade assimilated reanalysis winds (OWI) did not appreciably improve model performance compared to the blended GAHM+NAM meteorology for Matthew. Calculated water level differences ranged from 0.2 to 0.6 m, and were typically twice as large for the GAHM simulations as for the GAHM+NAM and OWI simulations. Results from the DA simulations were very favorable, with measures of model surge bias changing from −0.22 to −0.79 m in the unassimilated simulations to −0.06 to 0.01 meters in assimilated simulations. Mean absolute errors were also reduced by 48% to 66% at assimilation sites and 16% to 45% at validation sites. Further, surge error statistics with the GAHM and GAHM+NAM meteorological fields were indistinguishable from those with the OWI reanalysis fields once DA was performed. Thus, the DA was able to account for the effect of far-field winds on water levels, absent in the simple GAHM parametric vortex model. It is not clear to what degree this accounted for antecedent conditions vs. remote forcing, though time series suggest both were at play. The ability to compensate for some of the error in the meteorological forcing is particularly useful for forecasting because high-quality, reanalysis meteorological fields are not available. The small increase in computational effort (typically a few percent) makes this method highly amenable to forecast applications.

The performance difference between the two assimilation methods for Matthew was equivocal, with both methods showing nearly identical error metrics. A simplified test case illustrated conditions in which solutions diverge, with differences being largely relegated to semi-enclosed waterbodies weakly tied to the open ocean. The merit of each method in such cases was argued to be case-dependent and tied to the expected accuracy of the difference field.

A generalized framework for assimilating water level data into storm surge models using continuous sequential DA has been proposed. Care has been taken to discuss implementation details as they pertain specifically to surge, and to provide recommendations on how to navigate these issues. In particular, discovery of the first guess error covariance due to unresolved drivers is intrinsically impaired by their absence from the model. This may support the use of multiple discrete difference fields corresponding to distinct scales of unresolved drivers.

### 9.1 Future work

Limitations of the proposed methodology are chiefly imposed by the need to specify the error covariance used in the assimilation. We were pleased that a very simple error covariance worked well and greatly reduced the water level errors in our case, but recognize the potential value of more sophisticated approaches. These should be able to address the current method’s largest shortcomings, while allowing for more observed data to be used. Ensemble optimal interpolation or EnOI (Oke et al., 2002, Evensen, 2003, Oke et al., 2009) may be a promising candidate here. EnOI is effectively a hybridization of ensemble Kalman filter and OI methods, whereby a stationary ensemble (e.g. a set of historical simulations) is used to prescribe the covariance, which can be made to vary over long (seasonal) time scales (Brasseur et al., 2006). Ideally, such an ensemble would come from a more complex model that includes our unresolved drivers, although even a 2D barotropic model should be able to recover first-order structures such as the disconnect between open-ocean and estuarine waterbodies. The combination of multiple discrete difference fields, corresponding to distinct scale of unresolved drivers, may provide useful aid as well. A better specification of the spatial structure of the elevation difference field may help determine the robustness of the PAP and CON methods.

Additional data sources would help constrain the DA. Satellite altimetry coverage is not guaranteed on the time scale of a storm surge event, and there are substantial difficulties in getting quality measurements within ∼50 km of the coast. However, continued improvements have allowed for measurements of storm surge increasingly close to the coast (Fenoglio-Marc et al., 2015, Madsen et al., 2007, Madsen et al., 2015). Efforts to bring online airborne measurements of water surface elevation (Wright et al., 2009) could also lead to more comprehensive water surface data in the immediate nearshore. Better data on offshore water levels will also allow for more physically-realistic closure of interpolated surfaces. This text has not discussed how velocity data might be incorporated in this system, nor their utility, though Peng and Xie (2006) indicated little gains in assimilating both compared to assimilating only water elevation in storm surge forecasting.

Improvements in knowledge of the system physics can aid the DA analysis. To this end, research into how hurricanes affect large-scale fluctuations such as the Gulf Stream (e.g. Todd et al., 2018) is of use. Comparison to a 3D ocean model’s water surface topography may help inform the structure of the error covariance. Improvements in model physics to reduce the need for the DA correction should also be pursued to whatever degree possible while maintaining compatibility with available computational resources.

## CRediT authorship contribution statement

**Taylor G. Asher:** Conceptualization, Data curation, Formal analysis, Investigation, Methodology, Project administration, Resources, Software, Supervision, Validation, Visualization, Writing - original draft, Writing - review & editing. **Richard A. Luettich Jr.:** Conceptualization, Funding Acquisition, Methodology, Project administration, Supervision, Writing - review & editing. **Jason G. Fleming:** Investigation, Software. **Brian O. Blanton:** Methodology, Software.

## Appendix A Supplementary data

MMC S1

## Footnotes

1 Hurricanes originating near the Cape Verde islands, more info here: http://www.aoml.noaa.gov/hrd/tcfaq/A2.html.

2 The observation error is assumed very small (1 mm) since NOAA water level gages are very accurate.

3 The lowpass filtering of our data suggests brief, episodic changes in correlation like the overtopping of a barrier island during a large storm surge likely need not be accounted for in the correlation matrix.

4 This is closely related to the motivation for the “fixed basis” form of the singular evolutive extended Kalman (SEEK) filter (Brasseur and Verron, 2006).

5 This would classify the approach a smoother, which utilizes both past and future data, as opposed to a filter.
