# Supplementary material for: Low frequency water level correction in storm surge models using data assimilation
Source: Ocean Model (Oxf). 2019 Dec;144:None. doi: 10.1016/j.ocemod.2019.101483 (PMC10624563; doi:10.1016/j.ocemod.2019.101483)
Supplement: MMC S1 [file mmc1.docx]

# Model Setup Details

For readers interested in further detail on the model setup, all input and output files have been made available online as referenced in the main text. However, there are also a handful of model parameters in the ADCIRC model that may be of particular interest to readers familiar with the model. This section addresses these. The default HSOFS fort.13 and fort.14 files were used.

- The minimum bottom friction (FFACTOR) is set to 0.0025.
- The Manning’s n value on the ocean floor is set to 0.02
- The wind drag cap is set to 0.0028.
- The wind drag law is Garratt.
- The wind scaling factor for OWI winds is set to 1.0.
- There aren’t any user-controllable coefficients for GAHM.
- The mesh vertical datum is Mean Sea Level

# Supplemental Figures and Tables


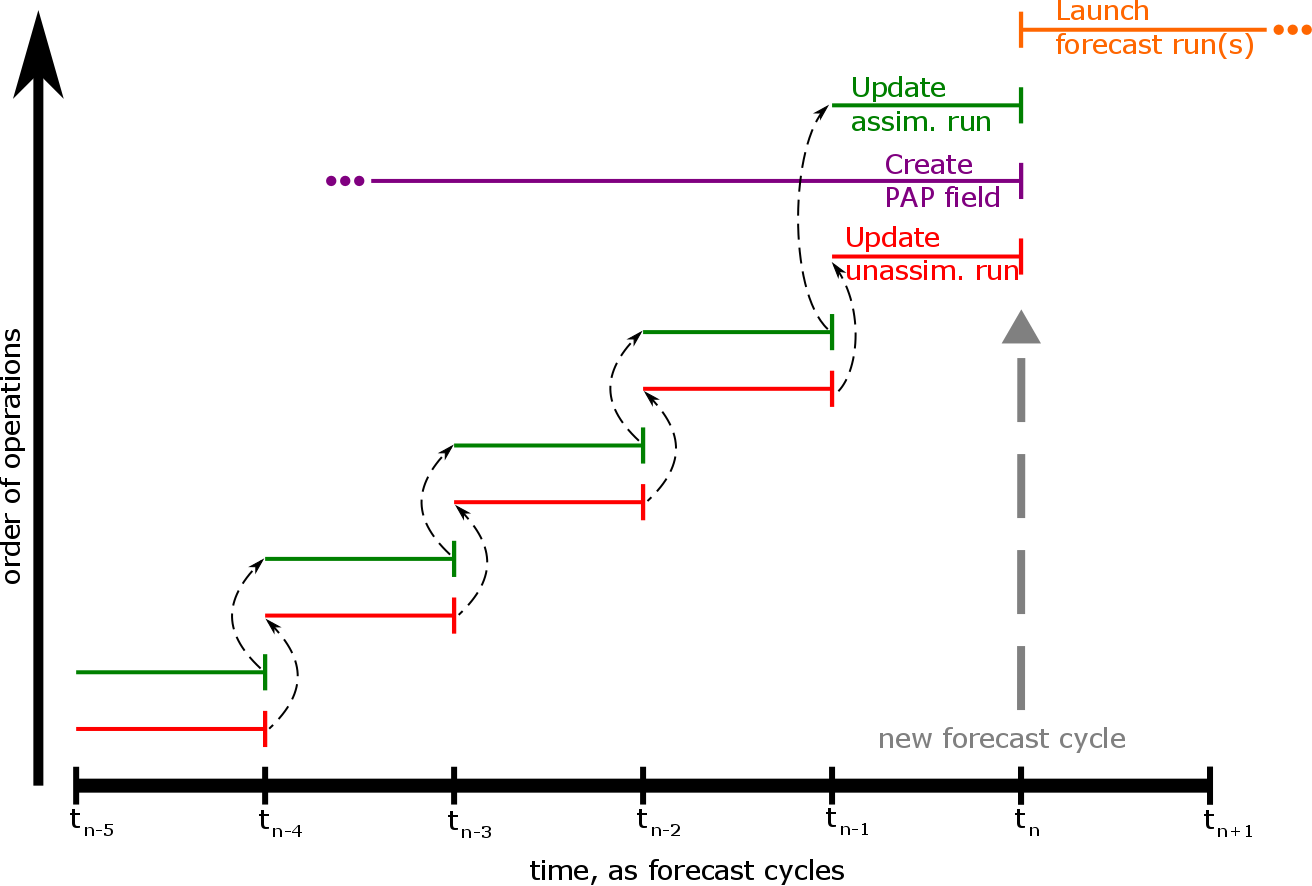


**Supplemental Figure 1**: Detailed schematic representation of DA system in an operational forecast context, with details shown for latest forecast cycle. Red denotes unassimilated simulations, green denotes assimilated simulations, curved dashed lines denote saving/restarting simulations across forecast cycles, Vertical tic marks denote the forecast cycle at which an action is taken; the order in which actions (operations) are taken is ordered from bottom to top of the schematic. Horizontal lines denote the temporal range over which an action’s data covers. Ellipses (...) denote items whose temporal extent is not specified explicitly here. Example: The following is a detailed explanation of steps taken by an operator (person or software performing the forecast steps) for the “current” *t_n_* forecast cycle.

1. Update unassimilated run:
   1. Acquire the latest meteorological information.
   2. Resume the unassimilated simulation (whose run state was previously saved at *t_n_*_-1_) running it over the time period (*t_n_*_-1_, *t_n_*].
   3. Save the model state at *t_n_*.
2. Create difference field:
   1. Acquire observed and modeled water level data over the time period [*t_n_* ‒ *T*, *t_n_*], where *T* is the averaging period described in the main article.
   2. Solve for Δ**ζ**^a^ using eq. (3): $\Delta\mathbf{\zeta}^{\text{a}}=\mathbf{K}\left\langle\mathbf{d}\right\rangle_{T}$
3. Update assimilated run:
   1. Resume the assimilated simulation (whose run state was previously saved at *t_n_*_-1_) running it over the time period (*t_n_*_-1_, *t_n_*]. Difference field forcing in the simulation Δ**ζ**^a^(*t*) is linearly interpolated between the analysis field created in this cycle $\Delta\mathbf{\zeta}^{\text{a}}(t_{n})=\Delta\mathbf{\zeta}_{n}^{\text{a}}$ and the field created at the last cycle $\Delta\mathbf{\zeta}^{\text{a}}(t_{n-1})=\Delta\mathbf{\zeta}_{n-1}^{\text{a}}$.
   2. Save the model state at *t_n_*.
4. Launch forecast run(s), whose initial state is taken from the assimilated run.

**Supplemental Figure 2**: CON method used for assimilation. Comparison of peak modeled and observed surges. Top: assimilation sites. Bottom: Validation sites. Left: colored lines are linear fits. Right: only gages whose observed peak surge exceeded 1 m are displayed to filter out non-event data.

**Supplemental Table 1**: CON method used for assimilation. Simulation identification and skill metrics for peak and time series water level data at assimilation sites and for peak data at validation sites, in meters.

|  | **Run ID** | GB | NB | OB | GA | NA | OA |
| --- | --- | --- | --- | --- | --- | --- | --- |
|  | **Met.** | GAHM | GAHM+NAM | OWI | GAHM | GAHM+NAM | OWI |
|  | **Assimilation** | no | no | no | yes | yes | yes |
| **Time series** | **MAE assim. set** | 0.40 | 0.22 | 0.21 | 0.12 | 0.09 | 0.08 |
|  | **RMSE assim. set** | 0.42 | 0.25 | 0.23 | 0.16 | 0.14 | 0.10 |
|  | **Bias assim. set** | -0.79 | -0.40 | -0.43 | 0.01 | 0.00 | -0.05 |
| **Peak surge** | **MAE assim. set** | 0.39 | 0.25 | 0.28 | 0.13 | 0.12 | 0.15 |
|  | **RMSE assim. set** | 0.43 | 0.29 | 0.32 | 0.19 | 0.17 | 0.19 |
|  | **Bias assim. set** | -0.39 | -0.22 | -0.25 | 0.00 | -0.03 | -0.07 |
|  | **MAE validation set** | 0.46 | 0.30 | 0.31 | 0.24 | 0.25 | 0.23 |
|  | **RMSE validation set** | 0.52 | 0.38 | 0.39 | 0.32 | 0.32 | 0.31 |
|  | **Bias validation set** | -0.42 | -0.22 | -0.22 | 0.03 | -0.01 | -0.04 |

**Supplemental Table 2**: Water level data sources; observed water levels were converted to mean sea level as needed using VDatum.

| Longitude | Latitude | Source | Station ID | Station Name | Observed peak water level (m MSL) |
| --- | --- | --- | --- | --- | --- |
| -75.0911 | 38.3283 | NOAA | 8570283 | Ocean City Inlet, MD | 0.95 |
| -75.9884 | 37.1652 | NOAA | 8632200 | Kiptopeke, VA | 1.14 |
| -76.2480 | 39.2133 | NOAA | 8573364 | Tolchester Beach, MD | 0.77 |
| -76.5770 | 39.2669 | NOAA | 8574680 | Baltimore, MD | 0.79 |
| -76.4816 | 38.9860 | NOAA | 8575512 | Annapolis, MD | 0.79 |
| -76.4508 | 38.3172 | NOAA | 8577330 | Solomons Island, MD | 0.85 |
| -76.4646 | 37.9954 | NOAA | 8635750 | Lewisetta, VA | 0.88 |
| -76.4788 | 37.2265 | NOAA | 8637689 | Yorktown USCG Training Center, VA | 1.11 |
| -76.3303 | 36.9470 | NOAA | 8638610 | Sewells Point, VA | 1.37 |
| -76.1133 | 36.9667 | NOAA | 8638863 | Chesapeake Bay Bridge Tunnel, VA | 1.30 |
| -76.3017 | 36.7783 | NOAA | 8639348 | Money Point, VA | 1.55 |
| -75.7460 | 36.1833 | NOAA | 8651370 | Duck, NC | 1.17 |
| -75.5531 | 35.7950 | NOAA | 8652587 | Oregon Inlet Marina, NC | 0.91 |
| -75.7020 | 35.2110 | NOAA | 8654467 | USCG Station Hatteras, NC | 1.85 |
| -76.6710 | 34.7200 | NOAA | 8656483 | Beaufort, NC | 1.20 |
| -77.7850 | 34.2133 | NOAA | 8658163 | Wrightsville Beach, NC | 1.49 |
| -77.9530 | 34.2275 | NOAA | 8658120 | Wilmington, NC | 1.76 |
| -78.9160 | 33.6550 | NOAA | 8661070 | Springmaid Pier, SC | 1.72 |
| -79.1867 | 33.3517 | NOAA | 8662245 | Oyster Landing (N Inlet Estuary), SC | 2.18 |
| -79.9220 | 32.7808 | NOAA | 8665530 | Charleston, Cooper River Entrance, SC | 1.94 |
| -80.9017 | 32.0367 | NOAA | 8670870 | Fort Pulaski, GA | 2.66 |
| -81.4670 | 30.6714 | NOAA | 8720030 | Fernandina Beach, FL | 2.27 |
| -81.4279 | 30.3990 | NOAA | 8720218 | Mayport (Bar Pilots Dock), FL | 1.75 |
| -80.5935 | 28.4158 | NOAA | 8721604 | Trident Pier, FL | 1.16 |
| -80.0342 | 26.6128 | NOAA | 8722670 | Lake Worth Pier, FL | 0.91 |
| -80.1617 | 25.7300 | NOAA | 8723214 | Virginia Key, FL | 0.66 |
| -81.1065 | 24.7140 | NOAA | 8723970 | Vaca Key, FL | 0.43 |
| -81.8079 | 24.6000 | NOAA | 8724580 | Key West, FL | 0.51 |
| -75.8344 | 36.3730 | USGS | 2043433 | CURRITUCK SOUND ON EAST BANK AT COROLLA, NC | 0.72 |
| -75.7683 | 36.0867 | USGS | 204347500 | CURRITUCK SOUND AT US 158 NR POINT HARBOR, NC | 0.98 |
| -76.4899 | 35.9604 | USGS | 208115501 | ALBEMARLE SOUND AT NC HWY 32/94 LEONARDS POINT, NC | 1.70 |
| -75.6221 | 35.8964 | USGS | 208117991 | ROANOKE SOUND AT US HIGHWAY 64/264 POND ISLAND, NC | 0.95 |
| -77.0640 | 35.5433 | USGS | 2084472 | PAMLICO RIVER AT WASHINGTON, NC | 1.39 |
| -77.0367 | 35.1026 | USGS | 2092576 | TRENT RIVER AT US HIGHWAY 70 AT NEW BERN, NC | 1.09 |
| -76.7368 | 34.7130 | USGS | 209270825 | BOGUE SOUND AT SR 1182 AT ATLANTIC BEACH, NC | 1.13 |
| -77.0633 | 34.6680 | USGS | 2092712 | BOGUE SOUND AT NC HIGHWAY 58 AT EMERALD ISLE, NC | 1.29 |
| -77.5493 | 34.4308 | USGS | 2093206 | INTRACOASTAL WTRWY AT NC HWY50/210 AT SURF CITY,NC | 1.27 |
| -77.7936 | 34.2170 | USGS | 2093222 | BANKS CHANNEL AT US HWY 74 WRIGHTSVILLE BEACH, NC | 1.61 |
| -77.9501 | 34.2518 | USGS | 210869230 | NE CAPE FEAR R AT US HWY 74/133 AT WILMINGTON, NC | 1.67 |
| -78.6558 | 33.8516 | USGS | 2110777 | AIW AT HIGHWAY 9 AT NIXONS CROSSROADS, SC | 1.99 |
| -79.8327 | 32.9243 | USGS | 217206962 | WANDO RIVER AT CAINHOY BELOW WANDO,SC | 2.14 |
| -81.0812 | 32.0810 | USGS | 21989773 | SAVANNAH RIVER AT USACE DOCK, AT SAVANNAH, GA | 2.43 |
| -81.3965 | 31.1315 | USGS | 2226180 | BRUNSWICK RIVER AT ST. SIMONS ISLAND, GA | 2.07 |
| -81.3000 | 29.9167 | USGS | 295500081180000 | TOLOMATO RIVER NEAR ST AUGUSTINE FL | 2.33 |
| -75.7718 | 36.2217 | Stormtide | NCDAR00008 | N/A | 0.87 |
| -75.7268 | 36.0179 | Stormtide | NCDAR12668 | N/A | 0.99 |
| -76.1846 | 35.9878 | Stormtide | NCTYR13548 | N/A | 1.21 |
| -75.6021 | 35.9096 | Stormtide | NCDAR12631 | N/A | 1.31 |
| -75.5492 | 35.7966 | Stormtide | NCDAR00005 | N/A | 0.85 |
| -75.5259 | 35.7675 | Stormtide | NCDAR12688 | N/A | 1.16 |
| -75.7704 | 35.6983 | Stormtide | NCDAR00010 | N/A | 0.81 |
| -75.4686 | 35.5833 | Stormtide | NCDAR12709 | N/A | 1.04 |
| -76.6147 | 35.5329 | Stormtide | NCBEA13648 | N/A | 1.04 |
| -76.3285 | 35.3935 | Stormtide | NCHYD00001 | N/A | 0.94 |
| -76.7482 | 35.3771 | Stormtide | NCBEA11728 | N/A | 1.17 |
| -75.5120 | 35.3502 | Stormtide | NCDAR00004 | N/A | 1.45 |
| -75.5576 | 35.2665 | Stormtide | NCDAR00002 | N/A | 2.01 |
| -76.5917 | 35.2449 | Stormtide | NCPAM13270 | N/A | 1.03 |
| -75.6353 | 35.2295 | Stormtide | NCDAR12748 | N/A | 2.19 |
| -75.6436 | 35.2247 | Stormtide | NCDAR18739 | N/A | 2.26 |
| -75.7028 | 35.2079 | Stormtide | NCDAR00001 | N/A | 1.85 |
| -76.8071 | 34.9675 | Stormtide | NCPAM13230 | N/A | 0.93 |
| -76.8107 | 34.9358 | Stormtide | NCCRV00003 | N/A | 0.96 |
| -76.4561 | 34.7969 | Stormtide | NCCAR12128 | N/A | 0.90 |
| -76.6085 | 34.7892 | Stormtide | NCCAR00012 | N/A | 1.13 |
| -76.6709 | 34.7173 | Stormtide | NCCAR12248 | N/A | 1.19 |
| -76.6815 | 34.6989 | Stormtide | NCCAR00007 | N/A | 1.33 |
| -76.7270 | 34.6967 | Stormtide | NCCAR12328 | N/A | 1.54 |
| -76.8957 | 34.6902 | Stormtide | NCCAR12409 | N/A | 1.25 |
| -77.1169 | 34.6875 | Stormtide | NCONS00001 | N/A | 1.33 |
| -76.5260 | 34.6848 | Stormtide | NCCAR00001 | N/A | 0.97 |
| -77.0957 | 34.6481 | Stormtide | NCCAR00005 | N/A | 1.38 |
| -77.3954 | 34.5762 | Stormtide | NCONS13128 | N/A | 0.98 |
| -77.3608 | 34.5437 | Stormtide | NCONS13168 | N/A | 1.01 |
| -77.3965 | 34.5029 | Stormtide | NCONS00002 | N/A | 1.60 |
| -77.6643 | 34.3665 | Stormtide | NCPEN13408 | N/A | 1.63 |
| -77.6282 | 34.3654 | Stormtide | NCPEN00003 | N/A | 1.96 |
| -77.7330 | 34.3113 | Stormtide | NCPEN00001 | N/A | 1.70 |
| -77.8114 | 34.2184 | Stormtide | NCNEW13008 | N/A | 1.70 |
| -77.8128 | 34.1892 | Stormtide | NCNEW00005 | N/A | 1.63 |
| -77.8875 | 34.0779 | Stormtide | NCNEW12928 | N/A | 1.69 |
| -77.8887 | 34.0585 | Stormtide | NCNEW12888 | N/A | 1.67 |
| -77.9190 | 34.0507 | Stormtide | NCNEW12908 | N/A | 1.73 |
| -77.9397 | 33.9613 | Stormtide | NCNEW00002 | N/A | 1.76 |
| -78.1446 | 33.9258 | Stormtide | NCBRU11890 | N/A | 1.97 |
| -78.2378 | 33.9217 | Stormtide | NCBRU11909 | N/A | 2.17 |
| -78.2675 | 33.9174 | Stormtide | NCBRU12008 | N/A | 2.09 |
| -78.0179 | 33.9170 | Stormtide | NCBRU12068 | N/A | 1.77 |
| -78.3738 | 33.9140 | Stormtide | NCBRU11908 | N/A | 2.17 |
| -78.1469 | 33.9128 | Stormtide | NCBRU11888 | N/A | 2.33 |
| -78.2972 | 33.9108 | Stormtide | NCBRU11868 | N/A | 2.39 |
| -78.0821 | 33.9036 | Stormtide | NCBRU11891 | N/A | 2.11 |
| -78.0188 | 33.8955 | Stormtide | NCBRU12048 | N/A | 1.79 |
| -78.4394 | 33.8951 | Stormtide | NCBRU00014 | N/A | 2.22 |
| -78.4360 | 33.8867 | Stormtide | NCBRU00012 | N/A | 2.44 |
| -78.5111 | 33.8821 | Stormtide | NCBRU11893 | N/A | 2.20 |
| -78.8167 | 33.7647 | Stormtide | SCHOR14332 | N/A | 2.02 |
| -78.8669 | 33.7406 | Stormtide | SCHOR14331 | N/A | 1.96 |
| -78.9369 | 33.6997 | Stormtide | SCHOR17781 | N/A | 1.92 |
| -78.9175 | 33.6594 | Stormtide | SCHOR17779 | N/A | 2.48 |
| -78.9739 | 33.6025 | Stormtide | SCHOR14328 | N/A | 2.67 |
| -79.0856 | 33.5619 | Stormtide | SCGEO14325 | N/A | 2.04 |
| -79.0314 | 33.5267 | Stormtide | SCGEO14321 | N/A | 2.22 |
| -79.1808 | 33.5131 | Stormtide | SCGEO14317 | N/A | 1.22 |
| -79.3831 | 33.3619 | Stormtide | SCGEO14322 | N/A | 1.77 |
| -79.2944 | 33.3569 | Stormtide | SCGEO14319 | N/A | 1.71 |
| -79.6572 | 32.9400 | Stormtide | SCCHA14308 | N/A | 2.06 |
| -79.7883 | 32.7897 | Stormtide | SCCHA14301 | N/A | 1.95 |
| -79.9586 | 32.7844 | Stormtide | SCCHA14295 | N/A | 1.92 |
| -80.1069 | 32.7844 | Stormtide | SCCHA14228 | N/A | 1.89 |
| -79.8417 | 32.7717 | Stormtide | SCCHA14305 | N/A | 1.88 |
| -79.9739 | 32.7669 | Stormtide | SCCHA14298 | N/A | 1.83 |
| -79.8567 | 32.7622 | Stormtide | SCCHA14309 | N/A | 1.87 |
| -80.0136 | 32.7528 | Stormtide | SCCHA14304 | N/A | 1.89 |
| -79.9439 | 32.6625 | Stormtide | SCCHA14297 | N/A | 1.97 |
| -80.3414 | 32.6356 | Stormtide | SCCHA14300 | N/A | 2.06 |
| -80.4811 | 32.6128 | Stormtide | SCCOL14314 | N/A | 2.05 |
| -80.1964 | 32.5975 | Stormtide | SCCHA14310 | N/A | 2.00 |
| -80.7447 | 32.5417 | Stormtide | SCBEA14279 | N/A | 2.41 |
| -80.3392 | 32.4936 | Stormtide | SCCOL14313 | N/A | 2.07 |
| -80.5997 | 32.4836 | Stormtide | SCBEA14282 | N/A | 2.45 |
| -80.6697 | 32.4300 | Stormtide | SCBEA14138 | N/A | 2.31 |
| -80.4497 | 32.4031 | Stormtide | SCBEA14277 | N/A | 2.30 |
| -80.8369 | 32.3739 | Stormtide | SCBEA14292 | N/A | 2.36 |
| -80.4631 | 32.3425 | Stormtide | SCBEA14286 | N/A | 2.31 |
| -80.6717 | 32.3347 | Stormtide | SCBEA14290 | N/A | 2.31 |
| -80.9306 | 32.2894 | Stormtide | SCBEA14139 | N/A | 2.57 |
| -80.8139 | 32.2869 | Stormtide | SCBEA14285 | N/A | 2.48 |
| -80.7939 | 32.2311 | Stormtide | SCBEA14280 | N/A | 2.43 |
| -80.8633 | 32.2311 | Stormtide | SCBEA14284 | N/A | 2.62 |
| -80.7697 | 32.1767 | Stormtide | SCBEA14293 | N/A | 2.35 |
| -80.8086 | 32.1397 | Stormtide | SCBEA14287 | N/A | 2.46 |
| -80.8990 | 32.0207 | Stormtide | GACHA17824 | N/A | 2.67 |
| -80.8467 | 31.9926 | Stormtide | GACHA17815 | N/A | 2.71 |
| -81.0106 | 31.9577 | Stormtide | GACHA17861 | N/A | 2.31 |
| -81.2781 | 31.7700 | Stormtide | GALIB17857 | N/A | 2.02 |
| -81.5330 | 31.1854 | Stormtide | GAGLY17811 | N/A | 2.22 |
| -81.4284 | 31.1706 | Stormtide | GAGLY17810 | N/A | 2.13 |
| -81.4981 | 31.1481 | Stormtide | GAGLY17821 | N/A | 2.07 |
| -81.4258 | 31.0674 | Stormtide | GAGLY17807 | N/A | 2.19 |
| -81.4348 | 31.0210 | Stormtide | GAGLY17790 | N/A | 2.19 |
| -81.6878 | 30.7419 | Stormtide | GACAM17830 | N/A | 1.37 |
| -81.5494 | 30.7200 | Stormtide | GACAM17823 | N/A | 2.28 |
| -81.4608 | 30.5105 | Stormtide | FLDUV03108 | N/A | 2.35 |
| -81.4350 | 30.3241 | Stormtide | FLDUV17794 | N/A | 1.71 |
| -81.4207 | 30.2876 | Stormtide | FLDUV03114 | N/A | 1.64 |
| -81.4104 | 30.2106 | Stormtide | FLSTJ03115 | N/A | 1.68 |
| -81.3104 | 29.9493 | Stormtide | FLSTJ03118 | N/A | 2.18 |
| -81.2859 | 29.8858 | Stormtide | FLSTJ17848 | N/A | 2.42 |
| -81.2539 | 29.7624 | Stormtide | FLSTJ03125 | N/A | 2.48 |
| -81.2308 | 29.7182 | Stormtide | FLSTJ03126 | N/A | 2.68 |
| -81.2211 | 29.6800 | Stormtide | FLSTJ03129 | N/A | 2.33 |
| -81.1755 | 29.5594 | Stormtide | FLFLA03131 | N/A | 1.57 |
| -81.1260 | 29.4799 | Stormtide | FLFLA03134 | N/A | 2.33 |
| -80.9650 | 29.1465 | Stormtide | FLVOL03141 | N/A | 2.37 |
| -80.9244 | 29.0850 | Stormtide | FLVOL03143 | N/A | 2.26 |
| -80.9159 | 29.0314 | Stormtide | FLVOL03145 | N/A | 0.36 |
| -80.8775 | 29.0095 | Stormtide | FLVOL03146 | N/A | 1.95 |
| -80.7950 | 28.5534 | Stormtide | FLBRE03161 | N/A | 0.41 |
| -80.5194 | 27.9215 | Stormtide | FLBRE03160 | N/A | 1.06 |
| -80.3479 | 27.5275 | Stormtide | FLSTL03731 | N/A | 1.04 |
| -80.2898 | 27.4707 | Stormtide | FLSTL03727 | N/A | 1.31 |
| -80.3300 | 27.4665 | Stormtide | FLSTL03732 | N/A | 0.86 |
| -80.2488 | 27.3631 | Stormtide | FLSTL03729 | N/A | 0.89 |
| -80.2507 | 27.2927 | Stormtide | FLSTL17773 | N/A | 0.90 |
| -80.2221 | 27.2527 | Stormtide | FLMAR00009 | N/A | 1.16 |
| -80.1921 | 27.2449 | Stormtide | FLMAR17784 | N/A | 1.19 |
| -80.1660 | 27.1997 | Stormtide | FLMAR03735 | N/A | 1.15 |
| -80.1113 | 27.0376 | Stormtide | FLMAR03740 | N/A | 0.67 |
| -80.0426 | 26.8249 | Stormtide | FLPAL03587 | N/A | 0.93 |
| -80.0391 | 26.7712 | Stormtide | FLPAL03581 | N/A | 0.88 |
| -80.0494 | 26.6923 | Stormtide | FLPAL17786 | N/A | 0.90 |
| -80.1733 | 25.8477 | Stormtide | FLMIA03341 | N/A | 0.74 |

**Supplemental Figure 3**: Modeled and observed time series for all stations for the base case and pseudo atmospheric pressure (PAP) method simulations. PAP forcing is ramped in over the first 24 hours (10/02/16 to 10/03/16). Black titles denote stations used in constructing the difference field, grey titles denote validation stations. “G”, “O”, and “N” denote GAHM, OWI, and GAHM+NAM sources of meteorology, respectively. “B”, and “A” denote baseline (unassimilated) and assimilated simulations, respectively. The third (bottom) panel in each figure is the nonlinearity in the water level response to the PAP forcing. This is calculated as the assimilated simulation minus the unassimilated simulation minus the difference field.
